# A Validated LC–MS/MS Assay for the Simultaneous Quantification of the FDA-Approved Anticancer Mixture (Encorafenib and Binimetinib): Metabolic Stability Estimation

**DOI:** 10.3390/molecules26092717

**Published:** 2021-05-05

**Authors:** Mohamed W. Attwa, Hany W. Darwish, Nasser S. Al-Shakliah, Adnan A. Kadi

**Affiliations:** 1 Department of Pharmaceutical Chemistry, College of Pharmacy, King Saud University, P.O. Box 2457, Riyadh 11451, Saudi Arabia; mzeidan@ksu.edu.sa (M.W.A.); nalshakliah@ksu.edu.sa (N.S.A.-S.); akadi@ksu.edu.sa (A.A.K.); 2 Students’ University Hospital, The Pharmacy, Mansoura University, Mansoura 35516, Egypt; 3 Analytical Chemistry Department, Faculty of Pharmacy, Cairo University, Kasr El-Aini St., Cairo 11562, Egypt

**Keywords:** binimetinib, encorafenib, LC–MS/MS, metabolic stability assessment, P450 module

## Abstract

The concurrent use of oral encorafenib (Braftovi, ENF) and binimetinib (Mektovi, BNB) is a combination anticancer therapy approved by the United States Food and Drug Administration (USFDA) for patients with BRAFV600E/V600K mutations suffering from metastatic or unresectable melanoma. Metabolism is considered one of the main pathways of drug elimination from the body (responsible for elimination of about 75% of known drugs), it is important to understand and study drug metabolic stability. Metabolically unstable compounds are not good as they required repetitive dosages during therapy, while very stable drugs may result in increasing the risk of adverse drug reactions. Metabolic stability of compounds could be examined using in vitro or in silico experiments. First, in silico metabolic vulnerability for ENF and BNB was investigated using the StarDrop WhichP450 module to confirm the lability of the drugs under study to liver metabolism. Second, we established an LC–MS/MS method for the simultaneous quantification of ENF and BNB applied to metabolic stability assessment. Third, in silico toxicity assessment of ENF and BNB was performed using the StarDrop DEREK module. Chromatographic separation of ENF, BNB, and avitinib (an internal standard) was achieved using an isocratic mobile phase on a Hypersil BDS C_18_ column. The linear range for ENF and BNB in the human liver microsome (HLM) matrix was 5–500 ng/mL (*R*^2^ ≥ 0.999). The metabolic stabilities were calculated using intrinsic clearance and in vitro half-life. Furthermore, ENF and BNB did not significantly influence each other’s metabolic stability or metabolic disposition when used concurrently. These results indicate that ENF and BNB will slowly bioaccumulate after multiple doses.

## 1. Introduction

Melanoma is the most high-risk type of skin cancer. The relative survival rate (5 years) for patients with distant melanoma in the United States (US) is 23% [1]. In 2018, the National Cancer Institute estimated that 91,270 new melanoma cases were diagnosed, and more than 9300 patients died of the disease in the US alone [2]. Cancer researchers are actively developing new treatments to improve patient outcomes for advanced melanoma. Specifically, the introduction of unique agents has sharply altered the treatment landscape for patients with all stages of melanoma. Available systemic treatments for patients with advanced melanoma include monoclonal antibodies, such as nivolumab that targets programmed cell death protein 1, and ipilimumab, which targets cytotoxic T-lymphocyte antigen-4, as well as oral small-molecule drugs that inhibit BRAF or MEK proteins [3].

The mitogen-activated protein kinase (MAPK) signaling pathway has a vital role in melanoma progression [4]. This pathway’s activation generates a signal cascade that results in sequential phosphorylation and activation of MAPK kinases, such as rat sarcoma (RAS), and the serine/threonine kinases rapidly accelerating fibrosarcoma (RAF), extracellular signal-regulated kinase (ERK), and MAPK/ERK kinase (MEK). These kinases regulate various cellular activities involving cell migration, proliferation, differentiation, angiogenesis, and survival. This pathway’s abnormal signaling can result in uncontrolled cell transformation and growth [5] and various cancer types. Activating BRAF mutations are found in approximately 20% of mucosal melanomas and 50% of skin melanomas [6].

**Figure 1:**
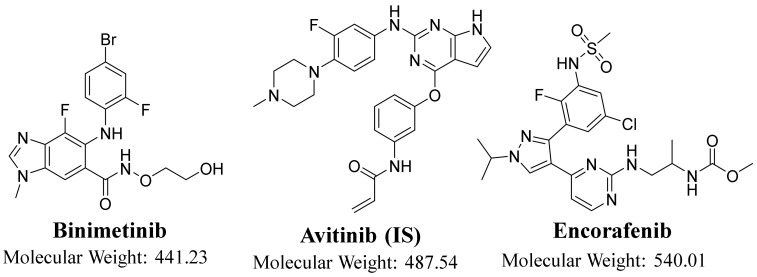
Chemical structures of binimetinib (BNB), avitinib (AVB), and encorafenib (ENF).

Binimetinib (Mektovi, BNB), a MEK inhibitor, and encorafenib (Braftovi, ENF), a BRAF inhibitor, are two orally bioavailable drugs established by Array BioPharma that are used for treating melanoma (Figure 1). ENF blocks the activity of a molecule called BRAF (mutated form). BNB stops a MEK molecule; MEK and BRAF are important protein molecules in cell growth regulation. BRAF V600K and V600E mutations signal for cells to begin abnormal growth and out-of-control splitting. These cells can be converted to a melanoma tumor, and approximately 50% of all melanomas have a BRAF mutation. MEK receives signals from BRAF and other molecules in the cell. In melanoma treatment, ENF stops the signaling pathway of the V600E-mutated BRAF molecule. BNB stops V600E- or V600K-mutated BRAF molecule signaling through the MEK molecule. Stopping BRAF and MEK molecules is more efficient than stopping BRAF alone [6,7]. The USFDA approved ENF combined with BNB (27 June 2018) for patients with BRAFV600E/V600K mutations and metastatic or unresectable melanoma [8]. The pivotal clinical trial, COLUMBUS, supported the approval of ENF combined with BNB for this indication [9]. The recommended oral doses are 450 mg ENF, once daily, and 45 mg BNB, twice daily [10].

Metabolism is considered one of the main pathways of drug elimination in human organs (responsible for the excretion of about 75% of known drugs) [11,12]. Therefore, metabolism can be the reason for problems with drug–drug interactions, bioavailability, and inter-individual differences [13,14], so it has a major influence in drug design [15]. Metabolic stability is considered one of the most important metabolism parameters that describes the vulnerability of compounds to metabolism. A metabolic stability study is an important step in the drug design pipeline when deciding on a drug’s acceptance or rejection [16]. Hence, it is important not only to characterize metabolites of drug candidates (studying their pharmacological activity and toxicity), but also metabolic stability of potential compounds, resulting in increasing drug acceptance rate thanks to performing many studies on increasing drugs’ metabolic stability [17]. Metabolic stability is expressed by various values (such as half-life or intrinsic clearance). Metabolic stability allows the assessment of how long a drug can be stable in a studied system, including in vitro and in vivo studies. Additionally, in silico approaches have been developed to predict in vitro half-life such as the StarDrop WhichP450 module software package [18,19,20].

Although several recent studies have discussed long-term severe adverse events after this combined therapy [21,22], the treatment of BRAF V600 melanoma combined with these two drugs appears advantageous compared with alternative treatments [8,23,24]. Therefore, developing a fast and reliable analytical method for the simultaneous estimation of BNB and ENF is essential. Metabolic stability studies are important tools for understanding drug metabolism; if a drug is moderately metabolized, it can have good bioavailability in vivo [25]. Additionally, studying the metabolic stability of the BNB and ENF combination may give insight into any mutual effect that the drugs’ metabolic stability may have.

Since both ENF and BNB are liver metabolized, it is, therefore, probable that the two drugs might affect each other’s metabolic stability [6,7,8]. This study’s primary aim is to investigate the effect of each component drug of the mixture on the metabolic stability of the other [25]. BNB and ENF were previously quantified in human plasma [26,27]. There is only one published article on the simultaneous analysis of BNB and ENF in pure pharmaceutical ingredients and formulations, but the linearity was 2–20 and 6–20 µg/mL for BNB and ENF, respectively, which does not reach the required limit for metabolic stability studies [28,29]. Thus, an appropriate and validated liquid chromatography–tandem mass spectrometry (LC–MS/MS) assay for the simultaneous detection and quantification of BNB and ENF in the biological matrix (human liver microsomes) was developed to achieve the required quantification limit [30,31,32]. Such a method is probably useful for calculating intrinsic clearance (CLint) and in vitro half-life (t_1/2_) [33] for metabolic stability assessments.

As an initial step, in silico metabolic vulnerability for the mixture’s two components was performed using the StarDrop WhichP450 module software package to predict such data. For the estimation of in vivo metabolic clearance rate from in vitro intrinsic clearance data, three basic models, dispersion, venous equilibrium, and parallel tube, could be used [34,35]. In this study, the metabolic stability of ENF and BNB involving intrinsic clearance and in vitro t_1/2_ in HLMs was computed according to the in vitro half-life approach, using the well-stirred model [36,37], as it is considered the most widely used model for in vitro drug metabolism experiments due to its simplicity. These in vitro parameters (in vitro t_1/2_ and intrinsic clearance) could be used for calculating different physiological parameters (in vivo t_1/2_ and hepatic clearance).

## 2. Results and Discussion

### 2.1. Results of In Silico ENF and BNB Metabolic Stability

**Figure 2:**
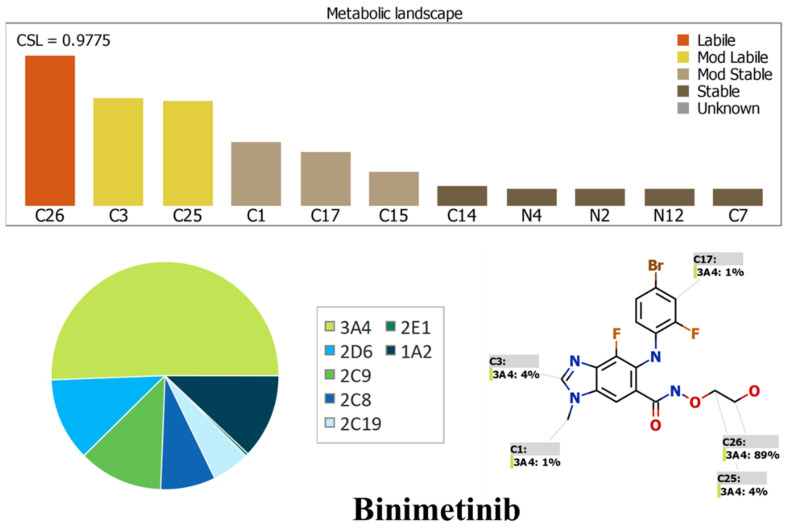
Proposed metabolic sites for BNB by StarDrop WhichP450™ module.

**Figure 3:**
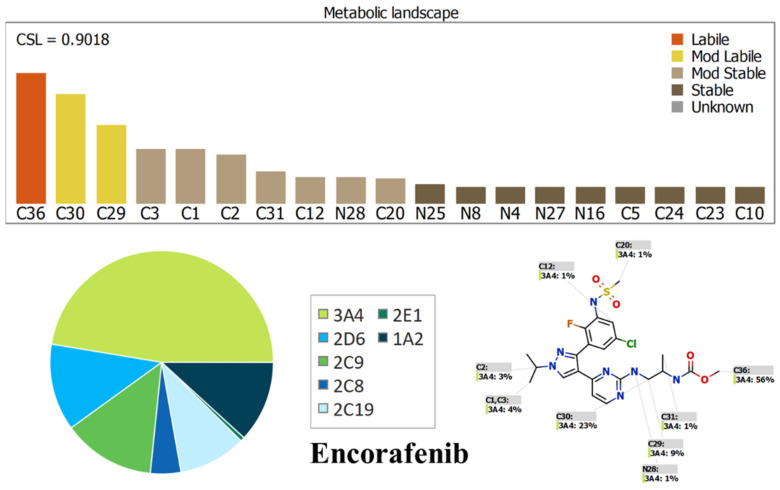
Proposed metabolic sites for ENF by StarDrop WhichP450™ module.

The metabolic landscapes for ENF and BNB indicate the lability of each site with respect to metabolism by CYP3A4 in absolute terms, to guide the optimization of chemical structure for improving metabolic stability. Composite site lability (CSL) values of BNB and ENF were 0.9775 and 0.9108, respectively, which indicates the lability of both drugs to metabolism by the liver. These values indicate that BNB and ENF are expected to be moderately metabolized in the liver, matching with the in vitro metabolic stability study. In BNB, C26 of the hydroxyl group was predicted to be the main labile site of metabolism, indicating the metabolic stability of BNB. The other metabolic soft spots are stable. In ENF, C36 of the methoxyl group and C29 and C30 of the 2-propanyl groups were predicted to be the main labile sites of metabolism, indicating the metabolic stability of ENF. The other metabolic soft spots are stable. The result from the WhichP450™ module, shown by the pie chart, was used for indication of the most likely cyp450 isoform that has a major role in BNB and ENF metabolism (Figure 2 and Figure 3). Cyp3A4 was found to have a major role in ENF and BNB metabolism. In silico results were supported by the experimental work that indicated the metabolic stability of ENF and BNB. According to pharmacokinetic information of encorafenib and binimetinib, CYP3A4 has a major role in encorafenib and binimetinib metabolism.

### 2.2. Results of In Silico BNB and ENF Structural Alert Sites and Toxicity Prediction

**Figure 4:**
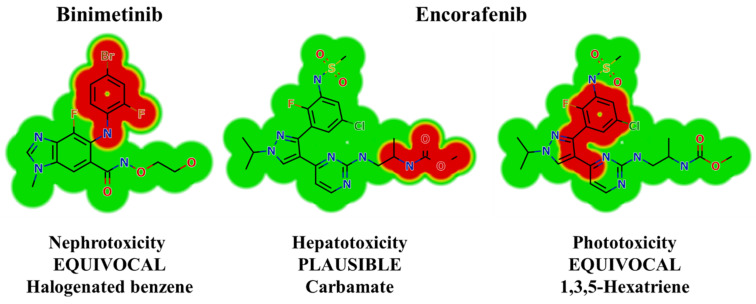
DEREK outcomes showing structural alerts with the proposed side effects of ENF and BNB. Red indicates the structural alerts.

In silico toxicity assessments of BNB and ENF were carried out using DEREK software. BNB shows structural alerts that cause proposed side effects, including nephrotoxicity (equivocal) due to halogenated benzene (Figure 4). ENF shows structural alerts that cause proposed side effects, including hepatotoxicity (plausible) and phototoxicity (equivocal) due to carbamate and 1,3,5-hexatriene, respectively (Figure 3).

### 2.3. HPLC–MS/MS Methodology

**Figure 5:**
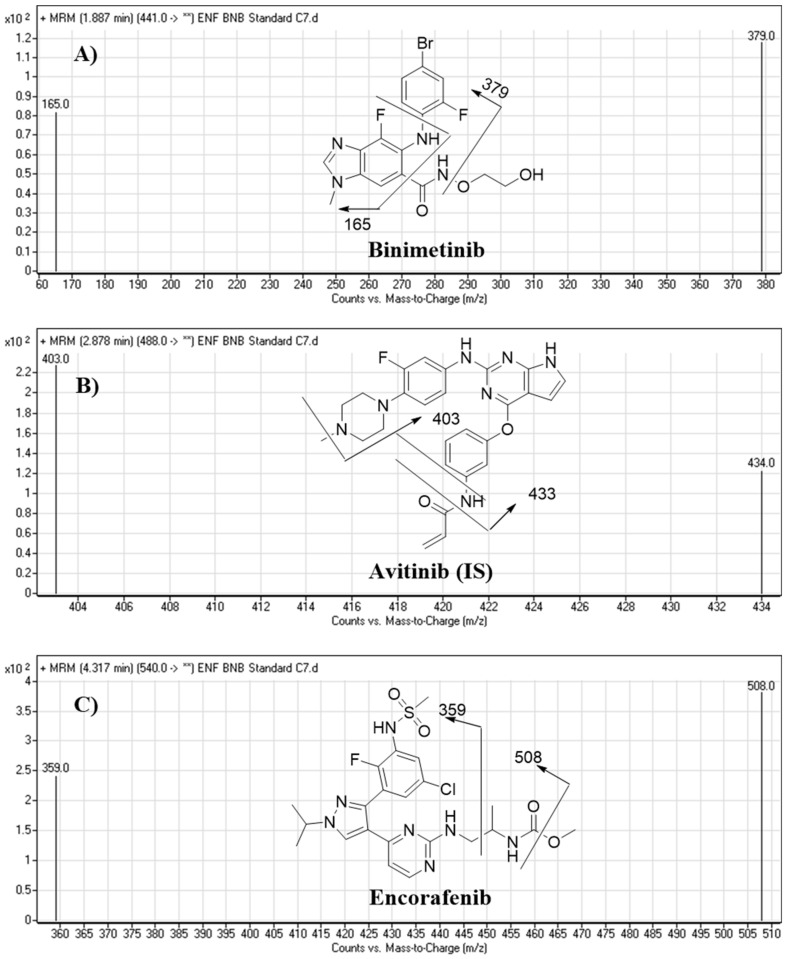
MRM mass transitions (parent to daughter ions) of BNB (**A**), AVB (IS) (**B**), and ENF (**C**) showing the selected daughter ions.

We optimized all chromatographic parameters involving the mobile phase constituents, mobile phase pH, and stationary phase. The pH of the aqueous mobile phase portion (10 mM ammonium formate) was optimized to 3.8 with formic acid, as higher pH values led to peak tailing and an unnecessary increase in elution time. The optimized mobile phase consisted of 38% aqueous and 62% ACN, as higher proportions of ACN reduced the resolution of the chromatographic peaks and lower proportions of ACN increased elution time. We tested the use of different stationary phases, such as HILIC columns, but such stationary phases were unable to retain or separate ENF, BNB, or AVB; however, good results were achieved using Thermo Scientific Hypersil BDS C_18_ columns. Figure 5 shows the MRM mass spectra for ENF, BNB, and AVB with their corresponding fragmentation patterns.

### 2.4. LC–MS/MS Method Validation

#### 2.4.1. Specificity

**Figure 6:**
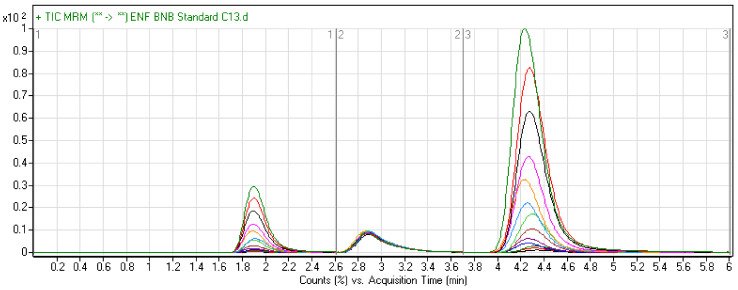
Overlaid MRM chromatograms of BNB at concentrations of 5.0–500.0 ng/mL at 1.89 min, AVB at a concentration of 100 ng/mL at 2.88 min, and ENF at concentrations of 5.0–500 ng/mL at 4.32 min. Standards and QC levels are exhibited in different colors: 500 (green), 400 (red), 300 (black), 200 (fuchia), 150 (orange), 100 (aqua), 80 (lime), 50 (maroon), 30 (purple), 20 (blue), 15 (olive), 10 (light red), and 5 (dark gray).

The chromatographic peaks of BNB, ENF, and AVB were eluted at different retention times. No overlapping peaks from the HLM blank were seen during the elution times of analytes. Chromatographic separation of ENF, BNB, and AVB was achieved with good resolution over a run time of 6 min (Figure 6). The MRM chromatograms showed good peak resolution of each analyte (ENF, BNB, and AVB) and an absence of peaks from the control HLM matrix at the corresponding retention times; these data confirm the specificity of the developed LC–MS/MS methodology.

#### 2.4.2. Sensitivity and Linearity

We established a linear range for the method from 5 ng/mL (LLOQ) to 500 ng/mL (upper limit of quantification; ULOQ) in the HLM matrix. Thirteen analyte standards were prepared, ten of them were used as calibration standards and three were used as quality controls. Six calibration curves of BNB and ENF were prepared on the first day of validation. The average from all data was used to establish a calibration curve for BNB and ENF. The regression equation for the ENF calibration curve was y = 2.3299x − 4.5808 (R^2^ = 0.9998). The standard error of the slope and intercept were 0.007089 and 1.501, respectively. The regression equation for the BNB calibration curve was y = 0.5287x − 2.2924 (R^2^ = 0.9997). The standard error of the slope and intercept were 0.0006829 and 0.1446, respectively. The lower limit of quantification was 5 ng/mL for both the ENF and BNB calibration curves. The relative standard deviation (RSD) values of six replicates for each standard concentration in the HLM matrix was less than 1.65% for ENF and less than 2.92% for BNB (Table 1). We performed back-calculations of the six calibration curves (calibration standards) and quality control (QC) samples of ENF and BNB in the HLM matrix, which showed good performance of the methodology. The results of the six calibration curves were used for linearity confirmation and intra-day validation. The relative standard deviation (RSD) values of calibration standards and quality controls in the HLM matrix were less than 1.65% for ENF and less than 2.92% for BNB (Table 1).

#### 2.4.3. Precision and Accuracy

The intra-day and inter-day precision and accuracy values were 0.45–2.60% and 96.11–100.25% for ENF, and 0.46–2.48% and 99.14–104.31% for BNB; these values are within acceptable ranges according to FDA guidelines for pharmaceuticals (Table 2).

#### 2.4.4. Matrix Effects and Extraction Recovery

The recoveries of ENF and BNB from the HLM matrix were 100.1% ± 1.1% and 99.73% ± 0.78%, respectively (Table 3). The HLM matrix did not influence the ionization of analytes. We found matrix effects of 99.12% ± 3.2% for BNB and 98.7% ± 2.1% for ENF. These results show that the HLM matrix exerts only minor effects on the ionization of ENF, BNB, and AVB (IS), and the extraction procedure using ACN shows a highly efficient procedure for extracting BNB and ENF for the HLM matrix.

#### 2.4.5. Stability

We evaluated the stability of ENF and BNB in deactivated HLM matrix (1 mg protein/1 mL phosphate buffer) under common laboratory storage conditions. Analyte stability evaluation was performed in different conditions: Room temperature for 8 h, three freeze–thaw cycles, storage at 4 °C for 24 h, and storage at −20 °C for 30 d. Measured values were 96.55–101.64% for ENF and 96.79–100.05% for BNB. Stability data for ENF and BNB are described in Table 4. We did not observe analyte degradation under the tested conditions. The stability data reveal that the LC–MS/MS method could be successfully used for assaying ENF and BNB without noticeable loss.

### 2.5. Metabolic Stability

**Figure 7:**
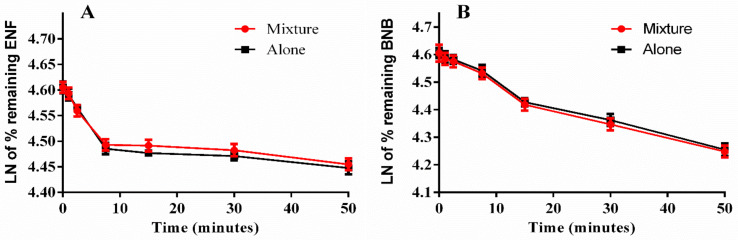
Metabolic stability curves of ENF alone and in a mixture with BNB (**A**) and BNB alone and in mixture with ENF (**B**).

The ENF and BNB levels in the HLM matrix were estimated using their peak area ratios in a linear calibration curve regression equation. Metabolic stability curves were constructed for ENF and BNB, both separately and mixed (Figure 7). The slopes of the linear regression equations of the constructed curves were used to calculate the in vitro t_1/2_. The regression equations were y = −0.0161x + 4.6059 for ENF alone (R^2^ = 0.9997), y = −0.0151x + 4.6042 for ENF mixed with BNB (R^2^ = 0.9906), y = −0.0115x + 4.6096 for BNB alone (R^2^ = 0.9757), and y = −0.0119x + 4.605 for BNB mixed with ENF (R^2^ = 0.9817) (Table 5).

The slope of each regression equation was used to estimate the in vitro t_1/2_ using the following equations:

(1) In vitro t 1/2=ln2slope

(2) In vitro t 1/2=0.693slope

Similarly, the CL_int_ of ENF and BNB was calculated following the in vitro t_1/2_ method (15) using the following equation:

(3) $${\mathrm{CL}}_{\mathrm{int},\;}=\frac{0.693}{{\mathrm{in}\;\mathrm{vitro}\;t\;}_{1/2}}.\;\frac{µL\;\mathrm{incubation}}{\mathrm{mg}\;\mathrm{microsomes}}$$

(4) $${\mathrm{CL}}_{\mathrm{int},\;}=\frac{0.693}{{\mathrm{in}\;\mathrm{vitro}\;t\;}_{1/2}}.\;\frac{1000\;}{1}$$

A validated LC–MS/MS method was developed for the simultaneous estimation of ENF and BNB that is characterized by sensitivity, rapidity (run time = 6 min), high recovery, and accuracy. This method was applied for studying the metabolic stability of ENF and BNB. The ENF and BNB metabolic stability calculations showed moderate CL_int_ (16.09 and 11.49 µL/min/mg) and in vitro t_1/2_ (43.1 and 60.3 min). Our calculations showed that when ENF and BNB are used concurrently, ENF is metabolized slightly slower, and BNB is metabolized slightly faster (Table 5). ENF is primarily metabolized through the CYP3A4 catalyzed phase I metabolism. In contrast, BNB is primarily metabolized by the UGT1A1 catalyzed conjugation and, to a lesser extent, by CYP1A2 and 2C19 catalyzed phase I metabolism [6,7,8], which supports our results of no significant effect on the metabolic stability of ENF and BNB when co-administered. These data and other parameters could also predict BNB and ENF in vivo pharmacokinetics using the Cloe PK simulation software. Furthermore, there does not appear to be any significant influence of ENF or BNB on each other’s metabolic stability or metabolic disposition when used concurrently; consequently, it is unnecessary to recalculate doses for concurrent use of ENF and BNB. From metabolic stability data, we can advise that plasma levels should be measured in cases where these drugs are used together since they can accumulate to toxic levels.

## 3. Materials and Methods

### 3.1. Chemicals and Reagents

HPLC-grade water was prepared by an in-house Milli-Q plus filtration system procured from Millipore (Millipore, Bedford, MA, USA). All reagents and solvents used were of analytical grade. Acetonitrile and formic acid were procured from Sigma-Aldrich (West Chester, PA, USA). ENF, BNB, and avitinib (AVB) were procured from Med Chem Express (Monmouth Junction, NJ, USA). Pooled male HLMs (product number: M 0567) were purchased from Sigma-Aldrich (West Chester, PA, USA) and stored at −70 °C until used. HLMs included a mixture of liver microsomes pooled from different individual male human donors.

### 3.2. In Silico Prediction of ENF and BNB Metabolic Vulnerability and Toxicity Using StarDrop WhichP450 and DEREK Modules

Before starting practical metabolic stability experiments, drugs should be tested for lability to drug metabolism in the liver. In silico metabolic stability for ENF and BNB was investigated using the WhichP450™ module of StarDrop software (Optibrium Ltd. Cambridge, MA, USA). Additionally, a literature review indicated that ENF and BNB are subjected to metabolism in the liver. Identification of ENF and BNB stability in terms of metabolism was provided by the composite site lability (CSL). The results from the WhichP450 module are shown by the pie chart and used for indications of the most likely cyp450 isoform that has a significant role in ENF and BNB metabolism. Screening for the predicted toxicity of ENF and BNB was performed using DEREK software that was also utilized to screen for their structural alerts.

### 3.3. LC–MS/MS Methodology

An Agilent 1200 HPLC (Agilent Technologies, Palo Alto, CA, USA) was used for chromatographic separation of analytes, and an Agilent 6410 QqQ triple quadrupole (Agilent Technologies, Palo Alto, CA, USA) equipped with ESI was used for the generation and detection of the eluted analyte ions. Agilent Mass Hunter software (Agilent Technologies, Palo Alto, CA, USA) was used for instrument data analysis and control. LC–MS/MS analytical parameters were optimized to achieve optimum separation of ENF, BNB, and AVB; AVB was used as an internal standard (IS) (Table 6). We used Agilent triple quadrupole mass analyzer operated in the positive ion mode with electrospray ionization (ESI) for mass analysis. Nitrogen (12 L/min) was used to dry the spray in the ESI source and the collision cell (60 psi) for dissociation studies. Direct injection was used to optimize all mass spectrometric analytical parameters to achieve the highest ion intensity. ESI source temperature (T) was set at 350 °C, while capillary voltage (V) was adjusted to 4000 V. Data acquisition was managed with the Mass Hunter software (Agilent Technologies, Palo Alto, CA, USA). The multiple reaction monitoring (MRM) mode of the QqQ was used to increase the selectivity and avoid interference of the HLM matrix in estimating ENF, BNB, and the IS, thereby elevating the LC–MS/MS method’s sensitivity [38,39,40].

### 3.4. Standard Solutions of ENF and BNB

ENF, BNB, and AVB are freely soluble in DMSO; accordingly, the first stocks were prepared for each of these compounds in DMSO at a 1 mg/mL concentration. We subsequently conducted serial dilution of the stock solutions with the optimized mobile phase to obtain stocks (S1) for ENF and BNB at a 100 µg/mL concentration. The S1 stocks were then serially diluted with mobile phase to obtain stocks (S2) for ENF and BNB at a 10 µg/mL concentration. The AVB stock was dissolved in DMSO to obtain a 100 µg/mL concentration that was then serially diluted with the mobile phase to obtain a stock (S3) of AVB at a 1 µg/mL concentration.

### 3.5. Preparation of Calibration Standards

BNB S2 (10 µg/mL) and ENF S2 (10 µg/mL) were diluted with a specific HLM matrix in phosphate buffer (1 mg protein/mL) to generate 13 standard levels: 5, 10, 15, 20, 30, 50, 80, 100, 150, 200, 300, 400, and 500 ng/mL. The 15, 150, and 400 ng/mL levels were selected as low quality control (LQ), medium quality control (MQ), and high quality control (HQ), respectively. AVB S3 (100 µL/mL) was used as an IS.

### 3.6. BNB and ENF Extraction from HLM Matrix

The sample extraction procedure’s target is to attain a high extraction percentage and decrease the biological matrix’s effect to increase the sensitivity and reliability of the established LC–MS/MS method. A low detection limit characterized the quantification of analytes using LC–MS/MS methodology compared with the HPLC method; therefore, the analytes were extracted successfully with a low matrix effect and good recovery. The ACN protein precipitation extraction procedure is a standard method for metabolic stability experiments [41,42,43,44]. Therefore, the protein precipitation method was chosen as it is characterized by short preparation time, fewer extraction steps, and simplicity if compared with solid extraction or liquid–liquid extraction. Therefore, ACN was used as a protein precipitation method to extract the BNB, ENF, and AVB analytes from the HLM matrix, followed by thermostatted centrifugation at 14,000 rpm and 4 °C for 15 min. Supernatants were filtered into 1.5 mL HPLC vials using 0.22 µm pore syringe filters. Five microliters of each sample were then injected into the LC–MS/MS for analysis.

Similarly, blank samples were prepared using phosphate buffer without the HLM matrix to confirm that the HLM matrix did not interfere with analyte retention times. The calibration curve of ENF was established by plotting the peak area ratio of ENF to AVB (y-axis) against the nominal concentration (x-axis) of ENF. The BNB calibration curve was established by plotting the peak area ratio of BNB to AVB (y-axis) against the nominal values (x-axis) of BNB. We used a linear regression equation to validate the developed method’s linearity; we calculated the slope, the coefficient of determination (R^2^), and intercept values.

### 3.7. Method Validation

The guidelines for analytical method validation of the FDA were followed for validation [45]. Method validation was performed for linearity, selectivity, sensitivity, precision, accuracy, extraction recovery, stability, and matrix effect [46]. We used the least-squares statistical method to compute the calibration curve equations (y = mx + b). Determination coefficient R^2^ was used to confirm the linearity of the constructed calibration curve.

#### 3.7.1. Specificity, Linearity, and Sensitivity

Analytical method specificity was investigated by the extraction of blank HLMs using the same extraction procedure. These extracts were then injected into the LC–MS/MS system and tested for any interference peaks for the retention time of BNB, ENF, and AVB.

Linearity and sensitivity of the developed analytical method were assessed using 12 calibration curves of BNB and ENF prepared on the first day of validation. The average from all data was used to establish a calibration curve for BNB and ENF. We performed back-calculations of the 12 calibration curves (calibration standards and QC samples) of ENF and BNB in the HLM matrix, which showed good performance of the methodology. The results of the 12 calibration curves were used for linearity confirmation and intra-day validation.

#### 3.7.2. Accuracy, Precision, and Stability

The accuracy and precision of the proposed method were investigated inter- and intra-day. The intra- and inter-day accuracy and precision values were calculated according to USFDA guidelines [42]. The stability of ENF and BNB in different conditions, room temperature for 8 h, three freeze–thaw cycles, and storage at 4 °C for 24 h and at −20 °C for 30 d, were examined.

#### 3.7.3. Matrix Effect and Extraction Recovery

The recovery and matrix effects in HLMs were investigated using QC samples, including low quality control, medium quality control, and high quality control. The recovery of BNB and ENF from HLMs was determined by comparing the peak area ratio response of both analytes in the optimized mobile phase (A) and those after protein precipitation (B). The ratio of B/A × 100 is defined as the percentage recovery. The matrix effect was determined by dividing the response of the post-extraction spiked sample (B) to the extracted BNB or ENF sample (C). Matrix effect equals (C/B × 100). A deviation in BNB and ENF by a maximum of 3.2% was considered an acceptable range, as recommended by the European guideline on bioanalytical method validation [47,48,49].

### 3.8. Metabolic Stability Assessment of BNB and ENF

BNB and ENF concentration during HLM incubation was adjusted to 1 µM to ensure that it was less than the Michaelis–Menten constant and constructed a linear relationship between the ratio of metabolism and incubation time. HLMs (1 mg/mL) in phosphate buffer were used to confirm the absence of nonspecific protein binding. We performed metabolic stability assessments for ENF and BNB by measuring the decrease in ENF and BNB concentrations after incubation with an HLM matrix. One micromole from ENF and BNB was incubated with HLM (1 mg protein in 1 mL phosphate buffer) in triplicate. The metabolic reaction medium was phosphate buffer (pH 7.4) containing 3.3 mM MgCl_2_. The metabolic mixture was pre-incubated in a 37 °C water bath for 10 min for temperature conditioning. NADPH (1 mM) was used for the initiation of the metabolic reaction, after which 2 mL ACN was used to terminate the reaction at specific time intervals (0, 1, 2.5, 7.5, 15, 30, and 50 min), allowing us to establish metabolic stability curves for ENF and BNB. We conducted this metabolic experiment for ENF alone, BNB alone, and a mixture of ENF and BNB to evaluate the metabolic stability of BNB and ENF alone and combined. The proportion of BNB and ENF remaining either alone or combined was plotted versus incubation time. From this plot, time points in the linear range were selected to plot the natural logarithm (ln) of the remaining proportions of ENF and BNB versus time. The slope of the linear part indicated the rate constant for ENF and BNB disappearance that was used for in vitro t_1/2_ calculation using equation (1). Then, ENF and BNB CL_int_ values, either alone or combined, were calculated by applying the following Equation (3) [50,51,52].

## 4. Conclusions

We established a validated LC–MS/MS method for the simultaneous estimation of ENF and BNB. The established method is sensitive, rapid (run time = 6 min), has a high recovery, and is accurate. ENF and BNB were each characterized by a moderate CL_int_ (16.09 µL/min/mg for ENF and 11.49 µL/min/mg for BNB) and a moderate in vitro t_1/2_ (43.1 min for ENF and 60.3 min for BNB), revealing a moderate clearance of ENF and BNB from the blood by the liver. This indicates a probable good in vivo bioavailability, which corroborates the good oral bioavailability previously reported. Additionally, these results indicate that ENF and BNB will slowly bioaccumulate after multiple doses.

Furthermore, there does not appear to be any significant influence of ENF or BNB on each other’s metabolic stability or metabolic disposition when used concurrently; consequently, it is unnecessary to recalculate doses for concurrent use of ENF and BNB. We can advise that plasma levels should be measured in cases where these drugs are used together since they can accumulate to toxic levels. The experimental outcomes were supported by the in silico WhichP450™ module of StarDrop software (Optibrium Ltd. Cambridge, MA, USA). In silico toxicological studies for ENF and BNB were performed using DEREK software that revealed structural alerts and proposed side effects. Further drug discovery experiments can be performed depending on these outcomes, permitting a new series of drugs with increased metabolic stability.

## Figures and Tables

**Table 1:** ENF and BNB concentration data back-calculated from the HLM matrix calibration levels.

Concentration in ng/mL	BNB				ENF			
	Mean ^a^	SD	RSD %	Accuracy %	Mean ^a^	SD	RSD %	Accuracy %
5	5.72	0.13	2.27	114.35	5.70	0.04	0.63	113.94
10	10.09	0.36	3.58	100.93	10.56	0.06	0.57	105.57
15 (LQC)	14.98	0.24	1.60	99.84	15.24	0.14	0.94	101.63
20	19.46	0.36	1.86	97.29	20.17	0.35	1.75	100.86
30	29.62	0.32	1.08	98.75	30.80	0.18	0.57	102.66
50	49.48	1.41	2.85	98.96	49.87	0.76	1.52	99.75
80	82.15	2.40	2.92	102.69	79.06	1.30	1.65	98.83
100	98.21	1.36	1.39	98.21	97.41	0.77	0.79	97.41
150 (MQC)	148.71	1.51	1.02	99.14	149.30	0.83	0.55	99.53
200	200.18	1.42	0.71	100.09	199.68	1.47	0.74	99.84
300	303.91	5.47	1.80	101.30	302.84	1.00	0.33	100.95
400 (HQC)	396.97	2.22	0.56	99.24	400.59	3.46	0.86	100.15
500	500.44	2.89	0.58	100.09	498.75	2.08	0.42	99.75
% Recovery				100.84				101.61
SD				4.29				4.20

^a^ Average of the results of six calibrations and quality control standards.

**Table 2:** Intra-day and inter-day accuracy and precision.

HLM Matrix				Mean	SD	% RSD	% Accuracy
BNB	Conc. in ng/mL	15.00 (LQ)	Intra-day assay *	14.98	0.20	1.31	99.8
			Inter-day assay **	15.02	0.37	2.48	104
		150.00 (MQ)	Intra-day assay	148.71	1.24	0.83	99.1
			Inter-day assay	148.68	1.37	0.92	100
		400.00 (HQ)	Intra-day assay	396.97	1.81	0.46	99.2
			Inter-day assay	397.78	3.52	0.88	101
ENF		15.00 (LQ)	Intra-day assay	15.24	0.12	0.77	101
			Inter-day assay	15.16	0.39	2.60	96.1
		150.00 (MQ)	Intra-day assay *	149.30	0.67	0.45	99.5
			Inter-day assay	148.20	1.88	1.27	96.7
		400.00 (HQ)	Intra-day assay	400.59	2.83	0.71	100
			Inter-day assay	399.80	2.92	0.73	100

* Average of 12 replicates of day 1. ** Average of six replicates in three consecutive days.

**Table 3:** Recovery of QC samples in the HLM matrix.

Concentration (ng/mL)	HLM Matrix					
	BNB			ENF		
	15	150	400	15	150	400
Mean ^a^	15.09	148.56	398.35	15.15	148.40	400.35
Recovery (%)	100	99.1	99.6	101	98.9	100
SD	0.38	1.30	3.55	0.36	1.79	3.03
Precision (RSD %)	2.51	0.87	0.89	2.38	1.21	0.76

**Table 4:** Stability of ENF and BNB in the HLM matrix (1 mg/1 mL phosphate buffer) under different laboratory conditions.

	Nominal Concentration (ng/mL)	Mean (ng/mL)	Recovery %	Precision (RSD %)
BNB	Room temperature for 8 h	14.89 ± 0.2	99.25	3.07
	15			
	150	147.38 ± 2.74	98.25	1.70
	400	395.31 ± 4.14	98.85	1.23
	Three freeze–thaw cycles	14.75 ± 0.27	98.32	0.46
	15			
	150	145.18 ± 2.04	96.79	2.52
	400	389.91 ± 3.19	97.48	4.87
	Stored at 4 °C for 24 h	15.01 ± 0.42	100.05	2.80
	15			
	150	146.38 ± 3.99	97.59	2.73
	400	397.31 ± 4.89	99.33	1.23
	Stored at −20 °C for 30 days			
	15	14.51 ± 0.67	96.72	4.59
	150	146.58 ± 2.39	97.72	1.63
	400	395.11 ± 4.57	98.78	1.16
ENF	Room temperature for 8 h			
	15	15.25 ± 0.3	101.64	2.00
	150	150.83 ± 4.17	100.55	2.76
	400	405.84 ± 4.45	101.46	1.10
	Three freeze–thaw cycles			
	15	14.61 ± 0.26	97.37	1.80
	150	145.83 ± 3.93	97.22	2.70
	400	398.64 ± 3.95	99.66	0.99
	Stored at 4 °C for 24 h			
	15	14.51 ± 0.35	97.37	2.37
	150	143.43 ± 2.85	96.95	1.96
	400	392.84 ± 3.16	98.21	0.81
	Stored at −20 °C for 30 days			
	15	15.03 ± 0.28	100.18	1.84
	150	144.83 ± 3.90	96.55	2.69
	400	393.64 ± 3.05	98.41	0.77

**Table 5:** ENF and BNB metabolic stability parameters after incubation with HLMs.

Parameters	ENF		BNB	
	ENF Alone	ENF with BNB	BNB Alone	BNB with ENF
Regression equation ^a^	y = −0.0161x + 4.6059	y = −0.0151x + 4.6042	y = −0.0115x + 4.6096	y = −0.0119x + 4.605
Slope	0.0161	0.0151	0.0115	0.0119
t_1/2_^b^	43.1 min	45.9 min	60.3 min	58.2 min
CLint ^c^	16.09 µL/min/mg	15.09 µL/min/mg	11.49 µL/min/mg	11.89 µL/min/mg
R^2^ ^d^	0.9972	0.9906	0.9757	0.9817

^a^ Regression equation in the linear part of metabolic stability curve. ^b^ Half-life. ^c^ Intrinsic clearance. ^d^ Determination coefficient.

**Table 6:** LC–MS/MS methodology parameters.

Agilent 1200 HPLC		Triple Quadrupole 6410 QqQ	
Isocratic mobile phase	ACN (38%)	ESI source	Positive mode
	10 mM ammonium formate in H_2_O (62%) adjusted with formic acid to pH 3.8		Drying gas: N_2_ gas Pressure (60 psi) Flow rate (12 L/min)
	Injection volume: 2 μL		
	Flow rate: 0.2 mL/min		
Agilent Hypersil BDS-C18	Length 125 mm, fully porous particle size (3.0 μm) and internal diameter (2.0 mm)		Source temperature: 350 °C
			Capillary voltage: 4000 V
		Mode	MRM mode
		Collision cell gas	Nitrogen with high purity
Analytes	Binimetinib (BNB)	BNB MRM transitions	m/z 441 → m/z 379, FVa: 140 V, CEb: of 22 eV
			m/z 441 → m/z 165, FV: 140 V, CE: of 20 eV
	Encorafenib (ENF)	ENF MRM Transitions	m/z 540 → m/z 508, FV: 135 V, CE: 18 eV
			m/z 540 → m/z 359, FV: 140 V, CE: 20 eV
IS	Avitinib (AVB)	AVB MRM transitions	m/z 488 → m/z 433, FV: 145 V, CE: of 15 eV
			m/z 488 → m/z 403, FV: 145 V, CE: of 16 eV

^a^ Fragmentor voltage. ^b^ Collison energy.

## Data Availability

All data are available within the manuscript.

## Sample Availability

The samples of compounds are not available from the authors.
